# Isoniazid prophylaxis: highly effective but underutilised to prevent tuberculosis in people living with HIV

**DOI:** 10.1016/S2214-109X(22)00408-9

**Published:** 2022-10-11

**Authors:** Lara Vojnov, W D Francois Venter

**Affiliations:** a Global HIV, Hepatitis and STI Programme, World Health Organization, Geneva 1211, Switzerland; b Ezintsha, Faculty of Health Sciences, University of Witwatersrand, Johannesburg, South Africa

In 2021, nearly 28 million (73%) of all people living with HIV accessed antiretroviral therapy; however, there were over half a million deaths in 2021, of which tuberculosis is the leading cause and a major contributor to advanced HIV disease.^1^, ^2^ COVID-19 has led to clear declines in tuberculosis testing and treatment access with increased morbidity and mortality.^2^ The tools to diagnose tuberculosis in people living with HIV exist, now supported with lateral flow urine lipoarabinomannan assays and near point-of-care molecular technologies. However, isoniazid preventive therapy (IPT), a highly effective, 6-month, simple, safe, and cost-effective preventive therapy that is widely available to high-burden low-income and middle-income countries, remains grossly underutilised.^2^, ^3^, ^4^, ^5^

Since 2016, WHO has strongly recommended a treat-all approach for people living with HIV: early and immediate treatment regardless of an individual's CD4 cell count.^6^ In *The Lancet Global Health*, Jinyi Zhu and colleagues^3^ remodel this evolving order: is IPT cost-effective with increasingly healthier cohorts of people living with HIV initiating antiretroviral therapy? The authors found that implementing IPT at current coverage levels had health benefits and was cost-effective and would avert almost 13 000 disability-adjusted life years (DALYs) in this cohort. Unsurprisingly, increasing the coverage rate of IPT averted considerably more DALYs. Interestingly, implementing IPT for healthier populations initiating antiretroviral therapy had greater lifetime health benefits yet without significant cost increases, and limited drug resistance. The authors did not include the effect of providing IPT in reducing onward transmission of tuberculosis—an important public health consideration. This study found that intuition that the clinical and cost benefits of IPT would diminish with people living with HIV initiating antiretroviral therapy when healthier appears incorrect. The study focused on a large urban health-care facility in Tanzania, and although largely resourced by the US President's Emergency Plan for AIDS Relief and a non-governmental organisation (and therefore receiving a level of resourcing that not all public facilities or programmes might receive), appears fairly representative of a public programme in Tanzania and Africa managing high HIV and tuberculosis burdens. The authors varied their input assumptions to account for possible epidemiological and programmatic differences across settings, with no significant changes in the results.

Zhu and colleagues, and others, have yet again provided the significant clinical, health, and cost benefits of implementing IPT, even at currently low coverage rates.^4^, ^5^, ^6^ The need and urgency of scale-up has been known for decades—WHO has recommended IPT since 1998.^7^ Unfortunately, the coverage of IPT has stagnated at about 50% of people living with HIV who initiate antiretroviral therapy. Reasons that might explain this substantial and multifactorial failure include clinician resistance, concerns about drug resistance, and previously over-complex guidelines.^2^ Ideally, national HIV and tuberculosis programmes would work together to improve access and coverage of IPT to ensure all people living with HIV receive this preventive intervention when needed, including those with antiretroviral therapy failure or those re-entering care, but this has not yet happened.

This might require a better understanding of why IPT scale-up has stalled and how to re-energise HIV programmes; the challenges and gaps across the programme, including and especially among people living with HIV and those supporting their care, must be recognised. Researchers, modellers, and guideline writers can only do so much. If the mortality benefits offered by IPT were seen in a new antiretroviral drug, activists would be calling for immediate access, clinicians clamouring for guideline inclusion, and public health specialists insisting every donor programme tie country money allocation directly to targets.

Part of the problem might be a more philosophically troubling one: HIV programmes at scale have poorly integrated other forms of care, not just tuberculosis screening and prophylaxis initiation. Attempts to integrate similarly intuitively appealing programmes such as family planning, chronic disease screening (eg, blood pressure), mental health and cervical cancer evaluations, and social vulnerability assessments around poverty and gender-based violence, have been largely unsuccessful. This could become a major problem in the future, as people living with HIV are living longer lives and being afflicted by obesity and non-communicable diseases, similarly to uninfected populations, including in low-income and middle-income countries. Effective integration of services will require stronger, more deliberate support to busy, under-resourced primary care facilities. However, there might be deeper, more complex systemic issues that need solving.

Whatever the reasons, fully implementing IPT is a clear clinical benefit for the individual. Further, it is mutually advantageous and cost-effective for both HIV and tuberculosis programmes. People with HIV will continue to need IPT as a life-saving measure—it is beyond time to prioritise full implementation of this critical intervention.

WDFV reports grants from USAID, Unitaid, the South African Medical Research Council, National Institutes of Health, Bill & Melinda Gates Foundation, and ViiV Healthcare; personal fees and non-financial support from ViiV and Gilead Sciences; and personal fees from Mylan, Merk, Adcock-Ingram, Aspen, Abbott, Roche, Johnson and Johnson, Sanofi, and Virology Education. LV declares no competing interests.
